# A scoping review of autism research conducted in Central Asia: Knowledge gaps and research priorities

**DOI:** 10.1177/13623613231170553

**Published:** 2023-05-10

**Authors:** Rano Zakirova-Engstrand, Gulnoza Yakubova

**Affiliations:** 1 Stockholm University, Sweden; 2 University of Maryland, USA

**Keywords:** autism research, Central Asia, knowledge gaps

## Abstract

Very little is known about the status of autism research in Central Asia. The purpose of this scoping review was to examine the scope and focus of the peer-reviewed research studies conducted with autistic people and their families in five Central Asian countries—Kazakhstan, Kyrgyzstan, Tajikistan, Turkmenistan, and Uzbekistan. The electronic databases of EBSCO Host and Web of Science were used for systematic search of literature. Eleven articles that met eligibility criteria were included in the review and analyzed for topics and research areas outlined in the Interagency Autism Coordinating Committee Strategic Plan. Ten of these studies were conducted in Kazakhstan and one in Uzbekistan using qualitative, quantitative, and mixed-methods research designs. Five of the seven research areas—Diagnosis, Biology, Risk Factors, Services, and Treatment and Interventions—were represented in these studies, while there were no studies identified in the areas of Lifespan Issues or Infrastructure and Surveillance, highlighting a gap in research. None of the studies reported co-authorship of scientists from Central Asian countries. The review identified knowledge gaps and research needs to guide future research in autism in Central Asia to address the needs of autistic individuals and their families living in this region.

**Lay abstract:**

Very little is known about the status of autism research in Central Asia. Through the library databases, we identified and reviewed 11 scientific studies conducted with autistic people and their families in five Central Asian countries—Kazakhstan, Kyrgyzstan, Tajikistan, Turkmenistan, and Uzbekistan. Of the 11 studies, 10 were conducted in Kazakhstan and 1 in Uzbekistan. Within these limited number of research studies, different topics such as diagnosis, risk factors of autism, biology, and various service and intervention areas were addressed. We identified several knowledge gaps and research priorities to address the needs of autistic people, their families, and professionals in Central Asia.

The paucity of autism-related research conducted in low- and middle-income countries (LMICs) has been well documented in the literature, reflecting geographic, cultural, and socioeconomic disparities in the field (Durkin et al., 2015; Hahler & Elsabbagh, 2015; Tomlinson et al., 2014). For instance, much research in the field of autism has been mainly conducted in high-income English-speaking countries or in Western Europe (Clark et al., 2017; Tomlinson et al., 2014), although 80% of global population live in LMICs, including 95% of children with neurodevelopmental disabilities below the age of 5 years (Amaral et al., 2019; Reed et al., 2019). Lack of autism-related studies in developing, low-resource nations presents significant challenges to timely identification of autism spectrum disorders (ASD) and provision of services across the lifespan because the best scientific evidence may not be available in these countries, which contributes to inequalities in accessing diagnostic assessments and high-quality educational, healthcare, and social services to autistic persons worldwide (Durkin et al., 2015).

The role that researchers play in generating scientific evidence is crucial in shaping policy, practice, and public awareness regarding existing disparities (Mir et al., 2012). Previous research from non-Western countries showed very low awareness about autism among general public and healthcare professionals (Abubakar et al., 2016; Habib et al., 2017), which could be one of the reasons for why many autistic individuals and their families experience social stigma, discrimination, and isolation in their everyday lives (de Leeuw et al., 2020). By informing general public about ASD and other neurodevelopmental disabilities and by building capacity in professionals on existing evidence-based practices in assessment and interventions, research in low-resource countries can facilitate the development of strong knowledge base and foster societies’ positive views and attitudes toward autistic persons and their families, thus contributing to improvement of their health and well-being.

In response to the challenges that autistic persons encounter, the United Nations (UN, 67/82, 2012) adopted a resolution “Addressing the socioeconomic needs of individuals, families and societies affected by autism spectrum disorders, developmental disorders and associated disabilities” (UN). This document calls upon national governments to focus on “enhancing and increasing research expertise and service delivery, including through international collaboration, by training researchers, service providers as well as non-professionals, in early diagnosis and interventions within health and other relevant sectors” (p. 3). Furthermore, the World Health Organization (WHO) adopted a resolution on *Comprehensive and Coordinated Efforts for the Management of Autism Spectrum Disorders* (WHO, 2013), where national governments are urged “to promote sharing of best practices and knowledge about autism spectrum disorders and other developmental disorders; [. . .] to identify and address disparities in access to services for persons with autism spectrum disorders and other developmental disorders,” and “to promote context-specific research on the public health and service delivery aspects of autism spectrum disorders and other developmental disorders, strengthening international research collaboration to identify causes and treatments” (p. 3).

Parallel to these efforts, the autism research community have identified key research priorities to guide research in LMICs: there is a strong need to conduct epidemiological studies to provide systematic information on availability and accessibility of diagnostic assessments for autism and intervention services (Elsabbagh et al., 2012); a need for development of feasible and reliable screening instruments and well-designed intervention studies has been particularly emphasized (Barbaro & Hadler, 2016; Hastings et al., 2012). Findings from recent review studies of autism-related research conducted in various geographic regions show that these knowledge gaps still remain. For instance, a review of research conducted in 22 Arab-speaking countries showed very limited attention to research questions addressing services, treatment and interventions for autistic people, lifespan issues, and surveillance (Alnemay et al., 2017). A study by Franz et al. (2017) found that autism research conducted in the countries of sub-Saharan Africa revealed the low number of peer-reviewed studies and the absence of studies reporting prevalence of ASD, early intervention, or studies examining any aspects of autism in adults, among others. The recent systematic review and meta-analysis of epidemiological studies conducted in Asia reported the ASD prevalence in nine countries located in East Asia, South Asia, and West Asia (Qui et al., 2020). Unfortunately, this review did not identify any epidemiological studies conducted in countries situated in Central Asia, and, therefore, did not report data on the ASD prevalence in that part of Asia—the region located at the crossroads of Asia and Europe (Suleimenov, 2021). Nor our own search of literature could detect any review study covering similar aspects in autism research originated in the region. To address this gap in the literature, this article presents the state of the art in autism research conducted with autistic people and their families in Central Asian countries—the region described in the literature as “terra incognita, mysterious, exotic, enigmatic, and attractive” (Roche, 2018, p. 95) and the area that exists in a “double periphery” (Eickelman, 2002, p. 2), which has been neglected by researchers.

Geographically, Central Asia has been given varying definitions and territorial boundaries (Frank, 1992): it could denote the area from Afghanistan to Mongolia and from Xinjiang in China to the Black Sea, or it could refer to the five former Soviet republics Kazakhstan, Kyrgyzstan, Tajikistan, Turkmenistan, and Uzbekistan (Roche, 2018). In this study, Central Asia is defined as the region stretching from the Caspian Sea in the west to China in the east and from the Russian Federation in the north to Afghanistan and Iran in the south and includes five former Soviet states—Kazakhstan, Kyrgyzstan, Tajikistan, Turkmenistan, and Uzbekistan (McKee et al., 2002). Historically, these Central Asian countries—a home to 73,212,000 people (United Nations, Department of Economic and Social Affairs, Population Division, 2019)—have been closely tied to the Silk Road and share common culture, history, and languages (Mukhitdinova, 2015).

Central Asia is a landlocked region characterized by its multi-ethnic population with a mixture of nomadic and sedentary ways of lifestyles, and where Islam is the dominant religion. According to several authors, the civilization of the entire region is based on the Persian-Islamic tradition established in the eighth–ninth centuries, and which was later influenced by the Turko-Mongolian culture (Eickelman, 2002; Pomfret, 1995; Roy, 2005). In the 1920s–1930s, the region became an integral part of the Soviet Union, and five socialist republics were created forming Soviet Central Asia. Since then and until the Soviet Union disintegrated in 1991, Central Asia was part of the Soviet centrally planned economy (Pomfret, 1995) with the Russian language being lingua franca (Agadjanian & Nedoluzhk, 2022). With the breakup of the Soviet Union, and similar to Central and Eastern European countries, the newly independent Central Asian states began the process of transitioning to market economy (Yadav, 2018). Thus, from a regional perspective and historically, the Central Asian region presents a natural unit with important differences from other neighboring countries located in its proximity (Pomfret, 1995). For instance, Mongolia was part of China until 1921 and has Buddhism as its largest religion, whereas Afghanistan was never part of the Soviet Union or of the Council for Mutual Economic Assistance (Pomfret, 1995). Furthermore, although Central Asian countries have followed different political and economic pathways based on the availability of natural resources such as oil, gas, gold, water, and cotton, all five countries inherited similar infrastructure from the Soviet era, for example, in education (Batsaikhana & Dabrowski, 2017), special education (Makoelle, 2020), healthcare (Dominis et al., 2018), and mental healthcare (Aliev et al., 2021). Today, the former Soviet Central Asian states are characterized by high levels of childhood immunization (United Nations Children’s Fund (UNICEF), 2022) and high rates of adult literacy and secondary education enrollment for both girls and boys (United Nations Educational, Scientific and Cultural Organization (UNESCO), Institute for Statistics, 2017). Also, of relevance, these countries have the highest proportion of trained teachers compared to other LMICs (UNESCO, Institute for Statistics, 2019). However, research spending as a share of gross domestic product (GDP) in Central Asian countries was less than 0.15% in 2018, which was similar to Mongolia’s expenditure (0.1%), but was considerably less than in other neighboring countries, for example, Iran (0.83% in 2017), Russia (0.99%), and China (2.19%) (UNESCO, 2021).

## Aims and objectives

The aim of this study was to investigate the scope and focus of the published peer-reviewed literature in order to understand the extent of the available evidence in autism research originated in five Central Asian countries. We specifically examined (1) topics and research areas represented in autism research in each country, (2) research designs of conducted studies, and (3) the rate, trajectory, and geographic representation of ASD research conducted in the region. The study also aimed at identifying and analyzing knowledge gaps and research needs to guide future research in autism in the Central Asian region. This study can be seen as a first step toward future research partnerships with and among researchers in Central Asian states to meet needs of autistic persons and their families.

## Method

To address the aims of the study, we conducted a scoping review of the published literature. Scoping reviews, or scoping studies, are a type of approach to review research literature in a field of interest that allows to rapidly “map” the key concepts used in a specific research area, examine the range of evidence, summarize and disseminate research findings, and identify research gaps in the existing literature (Arksey & O’Malley, 2005). Scoping reviews differ from full systematic reviews in multiple ways: they tend to address broader research questions where many different research designs might be used; they do not typically assess the quality of included studies, and may not involve extensive data extraction (Arksey & O’Malley, 2005; Armstrong et al., 2011; Levac et al., 2010). Scoping reviews may be particularly useful in disciplines with emergent evidence where randomized controlled trials are lacking, and they allow reviewing study designs in both published and gray literature (Levac et al., 2010). Therefore, undertaking a scoping review was seen as an appropriate method of creating preliminary mapping of the literature on a specific topic in research publications that originated in the Central Asian region.

### Central Asian context

Scientific output in autism coming from Central Asian countries has not occurred until recently. There were no publications in autism-related topics from any of the Central Asian countries during the period of 1980–2010 (Office of Autism Research Coordination (OARC), National Institute of Mental Health and Thomson Reuters, Inc. on behalf of the Interagency Autism Coordinating Committee (IACC), 2012). While there could be several explanations for this (e.g. lack of autism diagnosis or misdiagnosis, lack of professionals to diagnose autism, limited infrastructure to support research in institutions of higher education), scientific productivity can also be closely linked to funding support and institutional infrastructure. The universities that support scientific productivity with time and funding are rare (e.g. Nazarbayev University, English-speaking, modern research university). Governments may also play a key role in supporting research infrastructure. For instance, one of these five countries, Kazakhstan adopted the Law on Science in 2011, which introduced a funding framework that allowed public research institutions and universities to use the funding to invest in research infrastructure, information and communication tools, and cover staff salaries (Mukhitdinova, 2015).

### Protocol and registration

For the search and selection of studies as well as for reporting and discussing findings, we followed the guidelines for reporting the Preferred Reporting Items for Systematic Reviews and Meta-Analyses extension for scoping reviews (PRISMA-ScR; Tricco et al., 2018) (Supplementary Table A). A review protocol for the study was not published.

### Eligibility criteria

Studies included in this review met the following inclusion criteria: (1) original empirical research focused on the topic of autism in any of the disciplines (e.g. education and special education, social work, speech therapy, medicine, psychology); (2) conducted in Central Asia (any one of the five countries); (3) participants were children, adolescents, or adults diagnosed with ASD and/or their family members; (4) peer-reviewed journal articles; and (5) published in English or Russian. We excluded studies if (1) a study was not focused on autism (e.g. discussed autism traits, but was not on the topic of autism per se), (2) majority of participant sample was not from Central Asia or majority of research was not conducted in Central Asia, and (3) full text of the study was not attainable. We also excluded review and meta-analyses articles, editorials, conceptual papers, letters to the editors, poster presentations, book chapters, and conference papers.

### Information sources

We used the database of Web of Science (WoS) and EBSCO Host databases for a systematic search of peer-reviewed articles: Academic Search Premier/Ultimate, Education Resources Information Center (ERIC), CINAHL, APA PsycINFO, and MEDLINE.

### Search strategy

We conducted the search using the following search terms and keywords with truncations: (autism OR Asperger’s OR pervasive developmental disorder OR autistic OR auti* OR developmental disab* OR ASD) AND (Central Asia* OR Central Asian OR Kazakhstan OR Kazakh* OR Kyrgyzstan OR Kyrgyz* OR Tajikistan OR Tajik* OR Turkmenistan OR Turkmen* OR Uzbekistan OR Uzbek*). We limited the search by languages of publication (English AND Russian) and publication type (peer-reviewed). The reason for choosing Russian was due to the fact that the researchers from the Russian Federation were either the first or the second research collaborators for all five Central Asian countries (Mukhitdinova, 2015) and both authors of this scoping review were fluent in Russian to extract data from studies in Russian language. We did not limit the dates of publication. The search for peer-reviewed literature was conducted between November 2021 and January 2022 with the last search of databases performed on 8 January 2022.

### Screening and study selection process

Study screening and selection was conducted in two steps. First, using the eligibility criteria, we independently reviewed titles and abstracts followed by full-text retrieval of relevant articles from the database WoS. Any disagreements between both authors/reviewers were resolved by discussions during Zoom meetings. As the second step, we undertook the study screening and selection process in the EBSCO Host databases. Any disagreements were discussed until consensus was reached.

### Data extraction and data analysis

Both authors independently reviewed each article and extracted data into an Excel form according to the following categories: publication year, author affiliations, purpose, research design, participants, research area according to the IACC Strategic Plan (2012), publication outlet and language, funding source, and country where research took place (one or more of five Central Asian countries). We used publication year to determine the rate and trajectory of published autism research over time and geographically in all five countries. We coded the author affiliations to understand the primary author’s country and whether that was (or not) different from the Central Asian country where research took place. We coded the publication outlet of studies to understand where autism research in Central Asia was being published. If the research took place in more than one country, we specified whether the research activities occurred in multiple countries or whether the authors were from multiple countries. To identify research areas and understand types of autism research conducted in Central Asia, the included studies were coded using seven research areas outlined and described by the IACC (OARC, National Institute of Mental Health and Thomson Reuters, Inc. on behalf of the IACC, 2012): (1) *Diagnosis*, (2) *Biology*, (3) *Risk Factors*, (4) *Treatments and Interventions*, (5) *Service*, (6) *Lifespan Issues*, and (7) *Infrastructure and Surveillance* (see Supplementary Table B for the definitions of research areas). Data were recorded in the data charting form as recommended in the literature (Peters et al., 2020; Pollock et al., 2021). Critical appraisal and risk of bias assessments were not performed as they are not required in scoping reviews (Pollock et al., 2021). The extracted data items are presented in Table 1.

**Table 1.** Included studies.

Citation	Study purpose	Design	Participants	Research area (per IACC-identified research areas)^a^	Journal name and publication language	Funding source (government; industry, NGOs; mixed)	Country where research took place
Amirbekova & Abdikerova, 2021	To explore the features of social interaction of children with ASD and explore the influence of parents’ efforts in changing the stigma of the society toward ASD	Qualitative, in-depth interviews	Parents of children with autism (*n* = 5)	Services	*Journal of Human Behavior in the Social Environment* (English)	NR	Kazakhstan
An, Kanderzhanova, et al., 2020	To explore parents’ experiences of using complementary and alternative medicine (CAM) and factors driving the use of CAM	Qualitative (cross-sectional, focus group interviews)	Parents of children with autism (*n* = 44)	Services	*Autism* (English)	Mixed (government and Nazarbayev University)	Kazakhstan
An, Chan, & Kaukenova, 2020	To examine parental perspectives on the availability and use of educational, healthcare, and social support services for their children with autism	Qualitative (focus group interviews)	Parents of children with autism (*n* = 17; 16 mothers, 1 father); children’s age 3–13; 3 girls, 13 boys	Services	*International Journal of Disability, Development and Education* (English)	NR	Kazakhstan
Kenzhegulova et al., 2019	To examine the prevalence of epileptiform activity via electroencephalography (EEG) testing among children with autism	Observational study (cross-sectional study design; descriptive quantitative methodology used in biomedicine/healthcare)	Children with ASD (*n* = 71; 23 girls and 48 boys)	Biology	*Neurosurgery and Neurology of Kazakhstan* (Russian)	NR	Kazakhstan
Markova & Sultanalieva, 2013	To explore parental perspectives on activism in creating inclusive educational opportunities and removing barriers for their children with autism and collaborations between governmental and non-governmental organizations	Qualitative (case study design: individual interviews, focus group interviews, and participant observation)	Parents of children with autism; education professionals, NGO leaders, sample size was not specified	Services	*The Journal of Social Policy Studies* (Russian)	NR	Kazakhstan
Mukhtarova et al., 2021	To determine the associations of glucose and insulin homeostasis in the polymorphisms of 10 genes and demographic variables as predictors of autism	Observational (retrospective case-control; analytic quantitative methodology used in biomedicine/healthcare)	A sample of children and adolescents with ASD (*n* = 101) and without ASD (*n* = 110); age range 8–15 years. In the ASD group, 82.2% were males and 44.6% females	Risk factors	*Electronic Journal of General Medicine* (Open Access; English)	Government (Ministry of Education and Science of the Republic of Kazakhstan)	Kazakhstan
Perfilyeva et al., 2019	To investigate an association of the rs1799836 genetic variant of the neurotransmitter-related gene—monoamine oxidase B—to symptoms of autism	Observational (case–control; analytic quantitative methodology used in biomedicine/healthcare)	262 children with ASD and their 126 typically developing siblings. In the ASD group: 77% males and 23 % females; in the control group—41% males; 59%—females	Risk factors	*Hindawi Disease Markers* (Open access; English)	Government (Ministry of Education and Science of the Republic of Kazakhstan)	Kazakhstan
Rakhymbayeva et al., 2021	To examine the use of individualized robot-assisted therapy to increase the social engagement of children with autism	Interaction design qualitative data: semi-structured interviews with parents and therapists	11 children with ASD (7 of them with ASD/ADHD) and their parents (interviews); 1 girl, 10 boys; 4–11 years; mean age 6.1 years (SD = 2.7 years); 8 children were non-verbal	Treatment and interventions	*Frontiers in Robotics and AI* (Open Access; English)	Nazarbayev University Collaborative Research Program grant	Kazakhstan
Somerton et al., 2021	To explore the knowledge and beliefs of professionals involved in the diagnosis of children with autism	Sequential mixed-methods design (a survey followed by individual semi-structured interviews)	45 survey participants and 26 interview participants—professionals involved in the diagnosis of autism (neurologists, psychiatrists, educational psychologists, clinical psychologists, and defectologists)	Diagnosis	Journal of Autism and Developmental Disorders (English)	Nazarbayev University	Kazakhstan
Sandygulova et al., 2019	(1) To explore the utility of a commercially available socially interactive robots using two humanoid NAO robots and explore practical ways of integrating robots into real-world settings (2) To improve children’s imitation and turn-taking skills using humanoid robot-assisted therapy	Interaction design with observations and semi-structured interviews (User experience (UX) design used in the design interaction field)	14 children with ASD and ADHD; aged 3–8 years; 12 boys and 2 girls; among them 6 had ADHD; 12 non-verbal and 2 verbal	Treatment and interventions	*Paladyn, Journal of Behavioral Robotics* (English)	NR	Kazakhstan
Usmanov et al., 2020	To examine the dynamics of clinical and neurological indicators of speech impairment following the transcranial micropolarization	Observational study (quantitative methodology used in biomedicine/healthcare)	46 children (34 boys and 12 girls) with elements of autism and with a speech impairment, attention deficit hyperactivity disorder, minimal brain dysfunctions (3–7 years old)	Treatment and interventions; biology	*International Journal of Pharmaceutical Research* (English)	NR	Uzbekistan

IACC: Interagency Autism Coordinating Committee; NGOs: non-governmental organizations; NR: not reported.

a Seven research areas: (1) Diagnosis, (2) Biology, (3) Risk Factors, (4) Treatments and Interventions, (5) Services, (6) Lifespan Issues, (7) Infrastructure and Surveillance.

### Community involvement

There was no community involvement in the study.

## Results

### Search results

**Figure 1.**
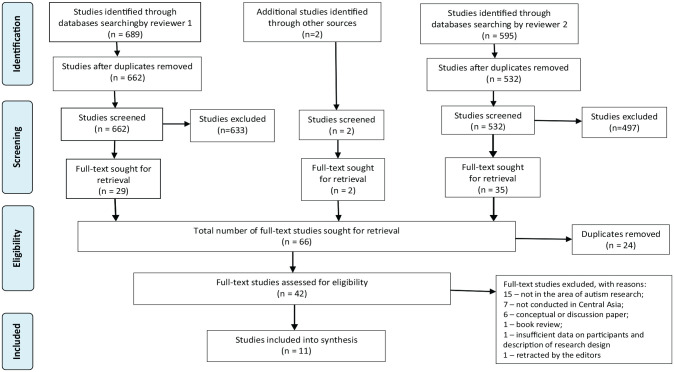
PRISMA-ScR flow diagram (Tricco et al., 2018).

The initial search in EBSCO Host databases, PsycINFO, and WoS database by the first author/reviewer resulted in 432, 76, and 181 entries, respectively, with a total number of 689. The search of studies by the second author/reviewer in EBSCO Host databases resulted in 418 entries, and in WoS database 177 entries, which resulted in a total of 595 entries. Figure 1 illustrates the identification, screening, and selection process. First level of screening included title screening. Then, both authors independently screened the abstracts of entries. We downloaded the full texts of 42 entries for further assessment, 4 of which were in Russian language. Both authors read the full texts of 42 articles and screened to determine the eligibility for inclusion in the scoping review. Eleven articles met the inclusion criteria. Thirty-one articles were excluded for several reasons (Figure 1).

### Inter-coder reliability

The inter-coder reliability was calculated by the number of agreements on each coding category per article divided by the total number of coded items. The initial inter-coder reliability for screening of full texts for inclusion was 95.24% and 100% agreement after discussions. When coding 11 included articles for the above-mentioned categories, the initial inter-coder agreement was 93.77% and 100% after discussions.

### Study characteristics

Table 1 presents characteristics of the included studies (*n* = 11). There were a total of 878 participants across 11 studies. The sample size in one study was not reported. Three studies included a total of 66 parents of autistic children as participants. There were 555 autistic children and adolescents as participants with a mean age range of 3–10 years and older in the studies that reported age or mean of age of participants across six studies (specific ages or age ranges were not reported in two studies). Typically developing children or siblings as participants in a comparison group comprised 236 children. Two studies included a variety of stakeholders involved in the education and care of autistic children but did not specify the sample size. The participants in these studies included education professionals, leaders of non-governmental organizations (NGO), parents, neurologists, psychologists, and others.

#### Topics and research areas

When analyzing the research areas of the studies according to the IACC Strategic Plan (OARC, National Institute of Mental Health and Thomson Reuters, Inc. on behalf of the IACC, 2012), four studies were identified to be in the area of *Service*, two studies in the area of *Risk Factors*, one study in the area of *Diagnosis*, two studies in the *Treatments and Interventions* category, one study in *Biology*. One study was categorized as falling simultaneously under two research areas—*Biology* and *Treatments and Interventions*. No studies were identified within the research areas of *Lifespan Issues* and *Infrastructure and Surveillance* (Table 1). The included studies addressed a wide range of aims: four studies examined parental perspectives and experiences on using supports and services (healthcare, educational, social) for their autistic children along with stigma and activism topics (Amirbekova & Abdikerova, 2021; An, Chan, & Kaukenova, 2020; An, Kanderzhanova, et al., 2020; Markova & Sultanalieva, 2013). Two studies focused on the user experiences when using robot-assisted therapy with autistic children (Rakhymbayeva et al., 2021; Sandygulova et al., 2019). One study focused on exploring the knowledge and beliefs of professionals involved in the diagnostic assessment for ASD among children (Somerton et al., 2021). Four studies were in the area of biomedical research with various aims, such as examining the prevalence of epileptiform activity via electroencephalography (EEG) testing (Kenzhegulova et al., 2019), examining the dynamics of clinical and neurological indicators of speech impairment following the transcranial micropolarization (Usmanov et al., 2020), and genetic and demographic variables as predictors of autism (Mukhtarova et al., 2021; Perfilyeva et al., 2019).

#### Research designs

The reviewed studies used a variety of research methods. Four of these studies used a qualitative methodology that involved in-depth and semi-structured individual or focus group interviews, observations, and site visits. Four other studies used a quantitative methodology with observational methods of cross-section and case–control designs in the field of biomedicine or healthcare. One study used a sequential mixed-methods design with a survey followed by individual semi-structured interviews. Two studies used the user experience design, specifically, the interaction design with observations and semi-structured interviews.

#### The rate, trajectory over time, and geographic representation of published research

Eleven studies were published during the years of 2013–2021: only 1 study was published in 2013, and 10 of these studies were published between 2019 and 2021. Nine were published in English and two in Russian in various peer-reviewed journals with four articles published in an open access format (Mukhtarova et al., 2021; Perfilyeva et al., 2019; Rakhymbayeva et al., 2021; Sandygulova et al., 2019). Ten studies took place with participants in Kazakhstan and one in Uzbekistan (Table 1). Five studies conducted in Kazakhstan reported funding sources to conduct the study. The funding involved both government and university funding sources. The study conducted with participants in Uzbekistan had the authors affiliated with the Tashkent Pediatric Medical Institute. Ten studies conducted in Kazakhstan had authors with various affiliations with majority of them conducted by authors at the Nazarbayev University (*n* = 6) and other universities and centers—Al-Farabi Kazakh National University, National Scientific Center for Maternity and Childhood, and NGO “Ashyk Alem” with an independent researcher in the United States (Markova & Sultanalieva, 2013). None of these studies reported co-authorship of scientists from various Central Asian countries.

## Discussion

In the present scoping review, we investigated the scope and focus of the academic, peer-reviewed literature in autism research conducted in five Central Asian countries—Kazakhstan, Kyrgyzstan, Tajikistan, Turkmenistan, and Uzbekistan. Only 11 studies met the inclusion criteria for the review. The analysis of these studies across the seven IACC Strategic Plan research areas showed that the research area with the most research publications was *Services*. There were no identified publications in the research areas of *Infrastructure and Surveillance* and *Lifespan Issues*, thus indicating a significant knowledge gap in those areas of autism research in Central Asia. The reviewed studies categorized as *Services* investigated primarily views and perspectives of parents of autistic children and of other key stakeholders (teachers, school principals, medical doctors) on a wide range of topics. These studies reveal parents’ ongoing concerns about timely and affordable access to many critical services necessary for timely diagnosis and support, for example, shortage of qualified professionals, negative attitudes toward children with disabilities in the society, and stigma associated with psychiatric assessment and psychiatric diagnosis. Parents also pointed to low awareness about autism among general public and healthcare and educational professionals—the finding supported by previous research from other LMICs (e.g. Abubakar et al., 2016; Bello-Mojeed et al., 2014; Shrestha & Santangelo, 2014). One of the included studies (Markova & Sultanalieva, 2013) is particularly informative as it describes the initiative of the parents of autistic children in Kazakhstan to establish a parent advocacy organization “Ashyk Alem” with the aim to support families of these children; it also mentions the organization’s involvement in the Central Asian network of parents of autistic children from other two countries in the region—Kyrgyzstan and Tajikistan. Lord and colleagues (2021) provide several examples on how the work of parent advocacy groups has played a crucial role in shaping policy and practice worldwide to ensure children’s rights to services—from earlier diagnosis to timely access to evidence-based treatments.

As previously noted, one of the results of this review was the absence of studies investigating lifespan issues which may indicate no research activity in the area of transition from secondary to post-school life or access to services for autistic youth and adults in Central Asia. Indeed, the participants in the included studies were mostly children aged 3–10 or older. Similarly, the review revealed the absence of prevalence studies from any of Central Asia states, which is consistent with previous research on the prevalence of autism in LMICs, including Asian countries (Elsabbagh et al., 2012; Qui et al., 2020). These results can be explained by differences in the definition of autism and its diagnostics in the former Soviet countries when autism in children was associated with childhood schizophrenia (Somerton et al., 2021; Simashkova et al., 2019). This view and conceptualization of autism might have an impact on identification and diagnostic assessment of autistic adults in Central Asia and, therefore, warrant further investigation for possible misdiagnosis of this population. Thus, our findings highlight a gap in research and practice regarding diagnostic assessment and early identification of autism due to lack of qualified professionals-diagnosticians and resources for conducting research in these areas.

Effective evidence-based treatments and intervention approaches are vital to supporting autistic individuals and their families (Lord et al., 2021), and therefore, it is one of the most prioritized areas in autism research (OARC, National Institute of Mental Health and Thomson Reuters, Inc. on behalf of the IACC, 2012). The present review revealed that research focusing on the development of treatments and interventions in Central Asian countries is given attention: two studies reported the use of robot-mediated interventions to improve social skills of autistic children. While this finding is encouraging, these studies are too few. Moreover, robot-mediated interventions for ASD have been described as very expensive and their use is usually limited to specialized clinical or research settings, and therefore, may not be feasible for use in LMICs (Kumm et al., 2022). In contrast, interventions based on accessible technologies and applications could be more beneficial for autistic individuals and their family members in daily lives (Divan et al., 2021). For instance, parents in one of the included studies shared their experiences on the effective use of WhatsApp for social support and communication with each other (An, Chan, & Kaukenova, 2020).

Our findings show that most of the included studies were conducted in Kazakhstan, while only one study was from Uzbekistan. These results reflect general trends in scientific output in various disciplines in Central Asia over time with Kazakhstan taking a leading role in the number of published research papers followed by Uzbekistan. For instance, Kazakhstan’s scientific output grew from 34.5% in 2005 to 55.8% in 2014 (Mukhitdinova, 2015), and in 2018, the country’s share of Central Asian scientific publications was 69% (Suleimenov, 2021). It is plausible that this trend—the overall increase of research output in various disciplines in Kazakhstan—can explain the growth in autism-related research publications from 2013 to 2021. Scientific productivity in Kazakhstan can also be closely linked to funding sources of the published research. The analysis of the available information from the funding acknowledgment section of the articles identified an important role that the Kazakh government or public research institutions played in supporting autism research in this country. Despite these efforts, overall investments of Kazakhstan and other Central Asian states in research still remain low (Suleimenov, 2021). As Mukhitdinova (2015) stated, “Progress in Central Asia is being hampered by the low level of investment in research and development” (p. 364), which may directly affect ASD research in these countries. Therefore, lack of funding can be seen as a major obstacle to higher level of research activity and research output on autism-related topics in the region. Another explanation to the dearth of ASD research in Central Asia is a lack of collaboration among researchers in different countries in the region. Alnemay and colleagues (2017) observed that the autism research output from the Arab-speaking countries that incorporates 22 members was the highest in Egypt and Saudi Arabia due to their close collaboration compared to other countries in the region. To tackle the current global imbalance in the knowledge, early detection, diagnosis and intervention of ASD, Barbaro and Halder (2016) call for researchers’ joint efforts to establish research networks through international collaboration.

### Strengths and limitations

This study is the first to map the existing evidence on types of autism research conducted in Central Asian countries in order to guide future research in the field. Another strength of the study is methodological: both authors in parallel and independently from each other conducted the searches, all phases of screening, and extraction of data for 100% of cases, which contributed to methodological rigor of the study. Both authors regularly met and discussed the aims, screening procedures, inclusion and exclusion criteria, and definitions for each coding category and content to be coded.

Along with strengths, this scoping review has several limitations. It is possible that additional primary studies conducted in Central Asia have been published after the present review was completed. Besides, we conducted our search of literature in several academic databases that might not have been accessible to Central Asian scientists. According to Suleimenov (2021), Kazakh researchers started subscribing to Thomson Reuter’s WoS in 2011 while their Kyrgyz and Uzbek colleagues got their subscriptions only in 2016. According to the author, researchers in Turkmenistan are not required to publish in international journals, which could be the reason for why we did not identify studies in autism-related topics from that country. It is possible that autism researchers in Central Asia publish their findings in publication outlets that are not indexed in large multidisciplinary databases of peer-reviewed, high-quality academic journals. In the future, similar reviews could be conducted in collaboration with colleagues from Central Asia who can facilitate access to local university databases of doctoral and master’s theses written in languages other than English or Russian.

## Conclusion and future directions

The results presented in this scoping review suggest that autism research in Central Asia is a very young but evolving field with the majority of studies conducted by researchers based in Kazakhstan. Low levels of research activities in the field of autism can be detrimental for the lives and well-being of autistic individuals and their families. There is an urgent need to strengthen research capacity in the region by building research infrastructure and invest in high-quality research in the areas of lifespan issues, services, and treatment and intervention. Investing resources in research can help identify important factors affecting the prevalence of ASD in Central Asian countries, such as the age of diagnosis, gender, co-occurring conditions, geographic location (urban/rural), socioeconomic background. Gained knowledge from such research could facilitate the development and implementation of appropriate interventions and timely access to individualized support and services to improve outcomes and quality of life of autistic individuals across the lifespan (Rice & Lee, 2017). Investment in capacity building of autism researchers in Central Asia is, therefore, paramount and should be considered as a priority.

Intervention researchers need to focus on establishing an evidence base by testing and evaluating the use of accessible and affordable technologies such as mobile and smartphones in various settings—home, schools, and clinics—to provide educational and healthcare services to autistic individuals and their family members and communicate with them (Divan et al., 2021). Another line of research needed in Central Asia could be feasibility studies of psychoeducational interventions for parents and extended family members (Ruparelia et al., 2016), and parent-mediated intervention packages that showed effectiveness in LMICs (Divan et al., 2021). Research focusing on cultural adaptations of existing manualized intervention packages is also necessary (Lord et al., 2021). However, this cannot be done without researchers’ close collaboration with autistic people, their families, and clinical and educational professionals who provide diagnostic and intervention services to children with developmental delays and autistic adults; therefore, it is important to use participatory research designs (Pickard et al., 2022).

The results of this study point to the pressing need to fund various types of autism research and research-related activities outlined above. Continued underinvestment in research in Central Asian countries in general (UNESCO, 2021) and the modest level of autism research output from these countries as shown in this study suggest that the governments of these countries need to put considerable efforts to increase financial investments in ASD research through universities, public research institutions, and government agencies. Involvement of business enterprises to support autism research initiatives should be encouraged. Furthermore, non-profit organizations involved in international development work, private research foundations, as well as disability advocacy groups might be interested in prioritizing funding autism research to advance the science and services in the region. Finally, there is a strong need to build collaboration between researchers and key community stakeholders in the region. Collaboration of international and local researchers and close collaboration of researchers from Central Asian countries are also necessary to address the needs of autistic individuals and their families in the region.

## Supplemental Material

sj-docx-1-aut-10.1177_13623613231170553 – Supplemental material for A scoping review of autism research conducted in Central Asia: Knowledge gaps and research priorities Click here for additional data file. Supplemental material, sj-docx-1-aut-10.1177_13623613231170553 for A scoping review of autism research conducted in Central Asia: Knowledge gaps and research priorities by Rano Zakirova-Engstrand and Gulnoza Yakubova in Autism

sj-docx-2-aut-10.1177_13623613231170553 – Supplemental material for A scoping review of autism research conducted in Central Asia: Knowledge gaps and research priorities Click here for additional data file. Supplemental material, sj-docx-2-aut-10.1177_13623613231170553 for A scoping review of autism research conducted in Central Asia: Knowledge gaps and research priorities by Rano Zakirova-Engstrand and Gulnoza Yakubova in Autism
