# Supplementary material for: A scoping review of autism research conducted in Central Asia: Knowledge gaps and research priorities
Source: Autism. 2023 May 10;28(2):342–54. doi: 10.1177/13623613231170553 (PMC10851649; doi:10.1177/13623613231170553)
Supplement: sj-docx-2-aut-10.1177_13623613231170553 – Supplemental material for A scoping review of autism research conducted in Central Asia: Knowledge gaps and research priorities [file sj-docx-2-aut-10.1177_13623613231170553.docx]

*Supplementary Table B:* *Research areas and corresponding critical questions and topics as per the* *IACC Strategic Plan*

|  | ***Research Area*** | ***Critical Questions*** | ***Topics Covered*** |
| --- | --- | --- | --- |
| 1. | Diagnosis | When should I be concerned? | Diagnostic and screening tests, early signs, biomarkers, symptomology, subgroups |
| 2. | Biology | How can I understand what is happening? | Neural systems, molecular pathways, developmental trajectory, cognitive studies, immune and metabolic pathways, neuropathology, biosignatures, computational studies, co-occurring conditions, sensory and motor functions |
| 3. | Risk Factors | What caused this to happen and can it be prevented? | Genetic risk factors, environmental risk factors, and the intersection of genetic and environmental risk factors, including epigenetics |
| 4. | Treatments & Interventions | Which treatments and interventions will help? | Pharmacological, behavioral, educational, complementary, dietary, occupational, sensory-based therapies, technology-based interventions and supports, model systems to identify molecular targets |
| 5. | Services | Where can I turn for services? | Utilization and access, evidence-based practices, cost-effective service delivery, practitioner training, family well-being, safety, community inclusion |
| 6. | Lifespan Issues | What does the future hold, particularly for adults? | Adult interventions, service needs, transition services, vocational rehabilitation, adult diagnosis |
| 7. | Infrastructure & Surveillance | What other infrastructure and surveillance needs must be met? | Surveillance and prevalence, research infrastructure, data tools, biobanks, research subject recruitment, research workforce development |

Adapted from the IACC/OARC autism spectrum disorder research publications analysis report (2012). <http://iacc.hhs.gov/publications-analysis/july2012/index.shtml>.
